# Supplementary material for: The effectiveness of Payments for Ecosystem Services at delivering improvements in water quality: lessons for experiments at the landscape scale
Source: PeerJ. 2018 Oct 23;6:e5753. doi: 10.7717/peerj.5753 (PMC6202973; doi:10.7717/peerj.5753)
Supplement: Table S1 — See Bottazzi et al. (2018) for more details of the Watershared agreements. [file peerj-06-5753-s001.doc]

|  | Level 1 | Level 2 | Level 3 |
| --- | --- | --- | --- |
| Eligible land | Forested land within 100m of a watercourse | Forested land within 100m of a watercourse | All non-agricultural and non-developed land |
| Principal required/ prohibited actions | Cattle removed/absent; no deforestation | Phased reduction/ removal of cattle; no deforestation | No land clearance |
| Value of incentives (US$/ha/year, equivalent in kind) | 10, plus 100 one-off payment on joining | 3 | 1 |
| Timescale for action | Immediately | Deforestation immediately; reduction/removal of cattle over 3 years | Immediately |
| Compliance monitoring | Yearly; in person; transects to inspect for cattle presence and signs of deforestation | Yearly; in person; transects to inspect for cattle presence and signs of deforestation | Classified satellite images |
| Total area under conservation (as of end 2014, ha) | 2206 | 1784 | 47683 |
