# Supplementary material for: The effectiveness of Payments for Ecosystem Services at delivering improvements in water quality: lessons for experiments at the landscape scale
Source: PeerJ. 2018 Oct 23;6:e5753. doi: 10.7717/peerj.5753 (PMC6202973; doi:10.7717/peerj.5753)
Supplement: Table S2 — It is important to note that while 129 communities were part of the RCT, due to logistical reasons water quality monitoring was only conducted at a subset of 120 of these at baseline and 118 at end-line. Some additional sites (not in the RCT) were monitored at end-line and included in the models exploring local predictors of water quality. The unit of analysis for the RCT analyses is communities but for the models exploring local predictors of water quality it is water systems (the combination of intake and tap). [file peerj-06-5753-s002.docx]

|  | Sites monitored at baseline in 2010 | Sites monitored in 2010 with comparable end-line (used in RCT differences-in differences analysis)* | Sites in RCT monitored at end-line in 2015 (used in RCT end-line only analysis) | Models exploring local predictors of water quality^***^ |
| --- | --- | --- | --- | --- |
| Intakes | 124 | 47 | 128 | 123 |
| Taps | 121 | 36 | 100 | 96 |
| Communities | 120 | 47 | 116** | - |
| Water systems | - | - | - | 124 |
| Sites | 246 | 83 | 228 | 219 |

* For the difference-in-differences analysis we are highly conservative and only include sites for which we are certain that the location at which we measured water quality in 2016 is the same as that at which water quality was measured in 2015.

** In two of the 118 RCT communities monitored at end-line, water was collected from a roof and so there is no mechanism by which cattle exclusion in the area can influence water quality. These sites were excluded from the analysis resulting in n=116.

*** Not all sites monitored in 2015 were included in the models exploring the local predictors of water quality, as some lacked a full set of predictors.

**Table S3. Variables monitored describing site condition or characteristics.**

| Disturbance | Categories | Location of monitoring |
| --- | --- | --- |
| Black sulphurous mud in intake | Present/Absent | Intake |
| Substrate in intake | Rocky/With Sand/With Mud | Intake |
| Filamentous algae in intake | Present/Absent | Intake |
| Faces in water or on riverbank | Present/Absent | 10m Transect |
| Faces in riparian forest | Present/Absent | 10m Transect |
| Litter | None; 1-5 items; 6-10 items; 11+ items | 10m Transect |
| Extractive activity | Present/Absent (if present, type) | 10m Transect |
| Cattle | Present/Absent | 10m Transect |
| Agriculture | Present/Absent | Intake |
| Forest cover | >80%; 50-80%; 10-50%; <10% | Intake |
| Forest connectivity | >75%; 50-75%; <50% | Intake |
| Fencing to prevent cattle access | Yes; No; No, but cattle cannot enter due to topography; Yes, but broken | Intake |
| Type of water source of intake | Stream; Spring; Roof rainwater collection | Intake |

**Table S4a. Model selection table for GLMMs exploring local predictors of 2015 *E. coli* concentration** **(biophysical variables only).** Codes are given in Table 2, water system N=124.

| Model | K | AIC | ωAIC | ΔAIC |
| --- | --- | --- | --- | --- |
| 7. 1\|Water System + SD + IC + ST + Tu | 5 | 919.49 | 0.5963 | 0 |
| 6. 1\|Water System + SD + IC + ST + C + Tu | 6 | 921.33 | 0.2379 | 1.84 |
| 5. 1\|Water System + SD + IC + ST + C + A + Tu | 7 | 923.31 | 0.08865 | 3.82 |
| 4. 1\|Water System + SD + IC + ST + C + A + Tu + S + pH | 9 | 925.50 | 0.02957 | 6.01 |
| 8. 1\|Water System + IC + ST + Tu | 4 | 926.90 | 0.01468 | 7.41 |
| 3. 1\|Water System + SD + IC + ST + C + A + Tu + Te + S + pH | 10 | 927.02 | 0.01381 | 7.53 |
| 9. 1\|Water System + SD + IC + Tu | 4 | 927.28 | 0.01218 | 7.79 |
| 2. 1\|Water System + SD + IC + ST + IS + C + A + Tu + Te + S + pH | 12 | 929.29 | 0.00444 | 9.8 |
| 11. 1\|Water System + SD + IC + ST | 4 | 932.03 | 0.001132 | 12.54 |
| 1. 1\|Water System + SD + IC + ST + IS + C + A + Tu + Te*S*pH | 16 | 932.53 | 0.000881 | 13.04 |
| 10. 1\|Water System + SD + ST + Tu | 4 | 933.81 | 0.000463 | 14.32 |

**Table S4b.** **Model selection table for GLMMs exploring local predictors of 2015 *E. coli* concentration (all variables)**. Codes are given in Table 2, water system N=119.

| Model | K | AIC | ωAIC | ΔAIC |
| --- | --- | --- | --- | --- |
| 16. 1\|Water System + SD + IC + ST + Tu + F | 7 | 917.34 | 0.3749 | 0 |
| 13. 1\|Water System + SD + IC + ST + Tu + CA + F | 8 | 919.09 | 0.1563 | 1.75 |
| 14. 1\|Water System + SD + IC + ST + Tu + ARA + F | 8 | 919.15 | 0.1517 | 1.81 |
| 7. 1\|Water System + SD + IC + ST + Tu | 5 | 919.49 | 0.1276 | 2.15 |
| 12. 1\|Water System + SD + IC + ST + Tu + CA + ARA + F | 9 | 920.92 | 0.06266 | 3.58 |
| 18. 1\|Water System + SD + IC + ST + Tu + ARA | 6 | 921.06 | 0.05843 | 3.72 |
| 17. 1\|Water System + SD + IC + ST + Tu + CA | 6 | 921.49 | 0.04693 | 4.15 |
| 15. 1\|Water System + SD + IC + ST + Tu + CA + ARA | 7 | 923.06 | 0.02149 | 5.72 |

**Table S4c. 95% confidence intervals of predictor coefficients in most likely model predicting *E. coli* concentrations in 2015** (model 16).

| Predictor (interpretation in model) | 2.5% | 50% | 97.5% |
| --- | --- | --- | --- |
| Intercept (log-transformed *E. coli* concentration) | 1.44 | 2.30 | 3.17 |
| Sediment (no disturbance of sediment) | -2.21 | -1.43 | -0.65 |
| Site type (tap compared with intake) | -1.10 | -0.67 | -0.23 |
| Intake category (spring compared with stream) | -1.84 | -1.25 | -0.67 |
| Turbidity (per 100 FAU) | 0.39 | 1.13 | 1.87 |
| Feces presence (in forest compared with absent) | -0.50 | 0.47 | 1.43 |
| Feces presence (in water compared with absent) | 0.25 | 1.91 | 3.57 |

**Table S5.** **95% confidence intervals of predictor coefficients in difference-in-differences analysis representing matched comparison of treatment and control community site *E. coli* concentration in 2015 while accounting for levels in 2010**.

| **Model coefficient** | **Value** | **Lower 95% value** | **Upper 95% value** |
| --- | --- | --- | --- |
| Log-transformed 2015  E. coli CFU concentration  in control sites (5ml equivalent) | -0.1 | -1.2 | 0.9 |
| Baseline effect of being a  treatment community site | -1.1 | -2.2 | 0.1 |
| Mean difference between  E. coli CFU concentrations  at sites in 2010 and 2015 | 0.2 | -1.2 | 1.7 |
| Difference in differences in E. coli  CFU concentrations between  2010 and 2015 between treatment  and control community sites | 0.9 | -0.9 | 2.7 |
| Community random effect | 1 | 0.6 | 1.9 |
| Sigma | 0.6 | 0.3 | 1.3 |

**Table S6.** **95% confidence intervals of predictor coefficients in model representing end-line analysis of *E. coli* concentration in 2015.**

| **Model coefficient** | **Value** | **Lower 95% value** | **Upper 95% value** |
| --- | --- | --- | --- |
| Log-transformed 2015  E. coli CFU concentration  in control sites (20ml equivalent) | 2 | 1.6 | 2.5 |
| End-line effect of being a  treatment community site | -0.3 | -0.8 | 0.1 |
| Tap compared  with intake | -0.6 | -1 | -0.2 |
| Spring intake compared  with stream intake | -0.8 | -1.3 | -0.4 |
| Water system random effect | 0.2 | 0 | 1285620.1 |
| Community random effect | 0.9 | 0.4 | 1.7 |
| Sigma | 12 | 7.7 | 18.8 |
