# Supplementary material for: The effectiveness of Payments for Ecosystem Services at delivering improvements in water quality: lessons for experiments at the landscape scale
Source: PeerJ. 2018 Oct 23;6:e5753. doi: 10.7717/peerj.5753 (PMC6202973; doi:10.7717/peerj.5753)
Supplement: Table S3 [file peerj-06-5753-s003.docx]

| Disturbance | Categories | Location of monitoring |
| --- | --- | --- |
| Black sulphurous mud in intake | Present/Absent | Intake |
| Substrate in intake | Rocky/With Sand/With Mud | Intake |
| Filamentous algae in intake | Present/Absent | Intake |
| Faces in water or on riverbank | Present/Absent | 10m Transect |
| Faces in riparian forest | Present/Absent | 10m Transect |
| Litter | None; 1-5 items; 6-10 items; 11+ items | 10m Transect |
| Extractive activity | Present/Absent (if present, type) | 10m Transect |
| Cattle | Present/Absent | 10m Transect |
| Agriculture | Present/Absent | Intake |
| Forest cover | >80%; 50-80%; 10-50%; <10% | Intake |
| Forest connectivity | >75%; 50-75%; <50% | Intake |
| Fencing to prevent cattle access | Yes; No; No, but cattle cannot enter due to topography; Yes, but broken | Intake |
| Type of water source of intake | Stream; Spring; Roof rainwater collection | Intake |
