# Supplementary material for: The effectiveness of Payments for Ecosystem Services at delivering improvements in water quality: lessons for experiments at the landscape scale
Source: PeerJ. 2018 Oct 23;6:e5753. doi: 10.7717/peerj.5753 (PMC6202973; doi:10.7717/peerj.5753)
Supplement: Table S4A — Codes are given in Table 2, water system N = 124. [file peerj-06-5753-s004.docx]

| Model | K | AIC | ωAIC | ΔAIC |
| --- | --- | --- | --- | --- |
| 7. 1\|Water System + SD + IC + ST + Tu | 5 | 919.49 | 0.5963 | 0 |
| 6. 1\|Water System + SD + IC + ST + C + Tu | 6 | 921.33 | 0.2379 | 1.84 |
| 5. 1\|Water System + SD + IC + ST + C + A + Tu | 7 | 923.31 | 0.08865 | 3.82 |
| 4. 1\|Water System + SD + IC + ST + C + A + Tu + S + pH | 9 | 925.50 | 0.02957 | 6.01 |
| 8. 1\|Water System + IC + ST + Tu | 4 | 926.90 | 0.01468 | 7.41 |
| 3. 1\|Water System + SD + IC + ST + C + A + Tu + Te + S + pH | 10 | 927.02 | 0.01381 | 7.53 |
| 9. 1\|Water System + SD + IC + Tu | 4 | 927.28 | 0.01218 | 7.79 |
| 2. 1\|Water System + SD + IC + ST + IS + C + A + Tu + Te + S + pH | 12 | 929.29 | 0.00444 | 9.8 |
| 11. 1\|Water System + SD + IC + ST | 4 | 932.03 | 0.001132 | 12.54 |
| 1. 1\|Water System + SD + IC + ST + IS + C + A + Tu + Te*S*pH | 16 | 932.53 | 0.000881 | 13.04 |
| 10. 1\|Water System + SD + ST + Tu | 4 | 933.81 | 0.000463 | 14.32 |
