# Supplementary material for: The effectiveness of Payments for Ecosystem Services at delivering improvements in water quality: lessons for experiments at the landscape scale
Source: PeerJ. 2018 Oct 23;6:e5753. doi: 10.7717/peerj.5753 (PMC6202973; doi:10.7717/peerj.5753)
Supplement: Table S4B — Codes are given in Table 2, water system N = 119. [file peerj-06-5753-s005.docx]

| Model | K | AIC | ωAIC | ΔAIC |
| --- | --- | --- | --- | --- |
| 16. 1\|Water System + SD + IC + ST + Tu + F | 7 | 917.34 | 0.3749 | 0 |
| 13. 1\|Water System + SD + IC + ST + Tu + CA + F | 8 | 919.09 | 0.1563 | 1.75 |
| 14. 1\|Water System + SD + IC + ST + Tu + ARA + F | 8 | 919.15 | 0.1517 | 1.81 |
| 7. 1\|Water System + SD + IC + ST + Tu | 5 | 919.49 | 0.1276 | 2.15 |
| 12. 1\|Water System + SD + IC + ST + Tu + CA + ARA + F | 9 | 920.92 | 0.06266 | 3.58 |
| 18. 1\|Water System + SD + IC + ST + Tu + ARA | 6 | 921.06 | 0.05843 | 3.72 |
| 17. 1\|Water System + SD + IC + ST + Tu + CA | 6 | 921.49 | 0.04693 | 4.15 |
| 15. 1\|Water System + SD + IC + ST + Tu + CA + ARA | 7 | 923.06 | 0.02149 | 5.72 |
