# Supplementary material for: The effectiveness of Payments for Ecosystem Services at delivering improvements in water quality: lessons for experiments at the landscape scale
Source: PeerJ. 2018 Oct 23;6:e5753. doi: 10.7717/peerj.5753 (PMC6202973; doi:10.7717/peerj.5753)
Supplement: Table S4C [file peerj-06-5753-s006.docx]

| Predictor (interpretation in model) | 2.5% | 50% | 97.5% |
| --- | --- | --- | --- |
| Intercept (log-transformed *E. coli* concentration) | 1.44 | 2.30 | 3.17 |
| Sediment (no disturbance of sediment) | -2.21 | -1.43 | -0.65 |
| Site type (tap compared with intake) | -1.10 | -0.67 | -0.23 |
| Intake category (spring compared with stream) | -1.84 | -1.25 | -0.67 |
| Turbidity (per 100 FAU) | 0.39 | 1.13 | 1.87 |
| Feces presence (in forest compared with absent) | -0.50 | 0.47 | 1.43 |
| Feces presence (in water compared with absent) | 0.25 | 1.91 | 3.57 |
