# Supplementary material for: The effectiveness of Payments for Ecosystem Services at delivering improvements in water quality: lessons for experiments at the landscape scale
Source: PeerJ. 2018 Oct 23;6:e5753. doi: 10.7717/peerj.5753 (PMC6202973; doi:10.7717/peerj.5753)
Supplement: Table S5 [file peerj-06-5753-s007.docx]

| **Model coefficient** | **Value** | **Lower 95% value** | **Upper 95% value** |
| --- | --- | --- | --- |
| Log-transformed 2015  E. coli CFU concentration  in control sites (5ml equivalent) | -0.1 | -1.2 | 0.9 |
| Baseline effect of being a  treatment community site | -1.1 | -2.2 | 0.1 |
| Mean difference between  E. coli CFU concentrations  at sites in 2010 and 2015 | 0.2 | -1.2 | 1.7 |
| Difference in differences in E. coli  CFU concentrations between  2010 and 2015 between treatment  and control community sites | 0.9 | -0.9 | 2.7 |
| Community random effect | 1 | 0.6 | 1.9 |
| Sigma | 0.6 | 0.3 | 1.3 |
