# Supplementary material for: The effectiveness of Payments for Ecosystem Services at delivering improvements in water quality: lessons for experiments at the landscape scale
Source: PeerJ. 2018 Oct 23;6:e5753. doi: 10.7717/peerj.5753 (PMC6202973; doi:10.7717/peerj.5753)
Supplement: Table S6 [file peerj-06-5753-s008.docx]

| **Model coefficient** | **Value** | **Lower 95% value** | **Upper 95% value** |
| --- | --- | --- | --- |
| Log-transformed 2015  E. coli CFU concentration  in control sites (20ml equivalent) | 2 | 1.6 | 2.5 |
| End-line effect of being a  treatment community site | -0.3 | -0.8 | 0.1 |
| Tap compared  with intake | -0.6 | -1 | -0.2 |
| Spring intake compared  with stream intake | -0.8 | -1.3 | -0.4 |
| Water system random effect | 0.2 | 0 | 1285620.1 |
| Community random effect | 0.9 | 0.4 | 1.7 |
| Sigma | 12 | 7.7 | 18.8 |
